# Comparison of Effects of Mothers' and Mozart's Lullabies on Physiological Responses, Feeding Volume, and Body Weight of Premature Infants in NICU

**DOI:** 10.3389/fpubh.2022.870740

**Published:** 2022-05-30

**Authors:** Hyo-Jin Shin, Jooyeon Park, Hye-Kyung Oh, Nahyun Kim

**Affiliations:** ^1^College of Nursing, Keimyung University, Daegu, South Korea; ^2^Department of Nursing, Daegu University, Daegu, South Korea

**Keywords:** premature infants, lullaby, physiological parameters, growth, neonatal intensive care unit (NICU)

## Abstract

**Objectives:**

The purpose of this study was to compare the effects of mothers' and Mozart's lullabies on physiological parameters, feeding volume, and body weight of premature infants in a neonatal intensive care unit (NICU).

**Methods:**

This study used a non-equivalent control group, non-synchronized design as a quasi-experimental study. Two intervention groups (recorded mother's lullaby and Mozart's lullaby) and a control group were formed from a total of 65 premature infants: a mother's lullaby group of 22 infants, a Mozart's lullaby group of 22 infants, and a control group of 21 infants not provided with any lullabies. Their physiological parameters included heart rate, blood pressure, respiratory rate, and O_2_ saturation. The infants' feeding volume and body weight were measured as indicators related to the growth of premature infants. The mother's and Mozart's lullabies were played on a speaker in an incubator for 15 min for 7 consecutive days per group.

**Results:**

There were significant differences in the mean difference before and after intervention in neonatal heart rate (χ^2^ = 45.03, *P* < 0.001), systolic pressure (F = 43.29, *P* < 0.001), diastolic pressure (χ^2^ = 33.01, *P* < 0.001), respiratory rate (F = 76.06, *P* < 0.001), and O_2_ saturation (χ^2^ = 40.82, *P* < 0.001) between the three groups. The mean differences of both mother's and Mozart's lullaby groups were significantly higher than those of the routine care group in all physiological parameters, and those of the mother's lullaby group was significantly higher when compared with the Mozart's lullaby group. In repeated-measures ANOVA, there was a significant interaction between time and group in feeding volume (F = 2.46, *P* = 0.041). However, body weight did not significantly differ in an interaction between time and group (F = 1.75, *P* = 0.151).

**Conclusion:**

This study showed beneficial effects of mother's lullaby and Mozart's lullaby on physiological parameters. Especially, the mother's lullaby was found to significantly improve all physiological parameters and feeding volume of premature infants in the NICU compared to Mozart's lullaby group. Therefore, we recommend the regular integration of the mother's lullaby into supportive care of premature infants in the NICU, as this intervention highlights the need for mothers to participate in their care.

**Trial Registration:**

ClinicalTrials.gov, identifier: KCT0004842 (https://cris.nih.go.kr).

## Introduction

In 2018, approximately 15 million babies were born preterm (before 37 completed weeks of gestation), and this number has been increasing (1). Prematurity is the leading cause of death of infants in the first month of life and is a factor in over 75% of pediatric deaths in the neonatal period (2). Premature infants do not adapt well to extrauterine life and can encounter problems related to respiration, low body weight, and hypothermic conditions (3). Consequently, this can lead to alterations in the quality and intensity of sensory stimuli of the infants and may adversely affect the normal maturation of their sensory system (4).

Additionally, premature infants are transferred immediately after birth to a neonatal intensive care unit (NICU) filled with various noises, such as mechanical sounds, conversations, and caregiver activities. In premature infants, noises can cause additional physiological stress (5), including changes in vital signs (6), sleep disturbance (7), and oxygen consumption (8). Exposure to inappropriate auditory stimuli during the early critical developmental period can result in long-lasting alterations in the connectivity and function of the sensory cortex (4). Such physiological stress can compromise growth and development (9–11).

Moreover, the interruption of the natural process of attachment or bonding with the mother while staying in the NICU should be carefully considered in terms of growth and development of the premature infants (12). Prematurity not only poses adverse effects on the psychological and biological development of infants, but also subjects them to stressors, such as emotional and physical detachment (13, 14). As such, separation-induced psychological stress could cause physiological instability, which is a sign of neonatal stress and a factor that hinders development (12, 13).

In Korea, a mother has close interaction with her baby during pregnancy through “Taegyo,” a custom for prenatal education consisting of various self-care behaviors that parents intentionally perform on a daily basis for their baby's physical and psychological growth and development (15). According to the literature, 90.3% of pregnant women in Korea practice Taegyo (16) wherein music activities, particularly singing and listening to music (especially lullaby, classical, traditional folkloric song), were the most common contents (17, 18). Additionally, other popular forms of “Taegyo” included reading books, telling fairy tales, talking to the fetus about everyday life, and calling the fetus nicknames (17). In this way, prenatal activities (Taegyo) are delivered to the fetus through the mother's voice, strongly affecting formation of mother-fetus attachment, as well as the physical, emotional, and intellectual development of the fetus (19, 20). However, due to the premature birth and continued separation from the mother, hospitalized preterm infants are exposed to considerably stressful situations, which can undermine their long-term development and mother-infant attachment or bonding (12, 13, 21). Therefore, it is necessary to minimize the psychological and subsequent physiological stress due to sensory deficits, noises, and separation from the mother in terms of supportive care.

In literature, music has been widely adopted as a favorable intervention for suitable auditory stimulation and relief of environmental, psychological, and physiological stresses in premature infants in the NICU. Based on studies on the effects of music therapy (MT) on premature infants, MT not only effectively stabilizes the heart rate (11, 22, 23), respiratory rate (11, 22–24), and behavioral state (11, 15, 22) of preterm infants, but also improves oxygen saturation (11, 22), feeding volume/ability/rate (11, 22, 23), and behavioral state (11, 22). Additionally, MT attenuates stress in infants, maternal anxiety (23), and length of NICU stay (22). However, the results of these studies were inconsistent and insufficient to confirm the effects of MT on the physiological and developmental state outcomes (23, 24). For example, some literature reports that MT has no significant effect on heart rate (24–26), respiratory rate (25–27), and oxygen saturation (23–25, 28). In addition, according to the recent meta-anaylsis study on the effect of MT, feeding volume and body weight were measured as the outcomes of MT intervention only in 2 of 13 RCT studies, respectively (23). Furthermore, the significant role of mother-infant attachment or bonding after birth in the growth and development of the child (19, 20) is rarely considered when using MT for premature infants in the NICU.

Among MT, the mother's voice and mother's song have been provided as optimal auditory stimulation to premature infants; both of them are known to have positive effects. In particular, the song sung by the mother, rather than the mother's voice, has a greater effect on the physiological and behavioral status of premature infants (29), and infants also prefer the mother's song to the mother's voice (30). Moreover, a mother's lullaby promotes emotional stability and infant development, and helps to forge a bond with the mother (31, 32). However, the effect of mother's lullaby on the physiological homeostasis of premature infants is still controversy (33), and studies on the effect of the lullaby on the feeding volume and body weight of the infants are lacking. Both feeding vloume and body weight are associated with growth and ultimately the development of premature infants (34–36).

Therefore, we examined the effects of the mother's lullaby, a song sung by a mother to her baby as a “Taegyo” during pregnancy, on the physiological stability, feeding volume, and body weight of premature infants during their stay in the NICU of a tertiary hospital in Korea. Moreover. we compared the effects of the mother's lullaby intervention with Mozart's lullaby, which is one of the most popular classical music pieces used in MT studies (37). We hypothesized that the lullaby in a mother's voice, being familiar to the infants since their fetal stage, would improve physiological homeostasis, feeding volume, and body weight of premature infants in the NICU and these effects would be higher than those of Mozart's lullaby.

## Methods

### Study Design

This study was conducted using a non-equivalent control group, non-synchronized, quasi-experimental design. The infants were selected from a single tertiary hospital located in D city, Korea. To control for the possibility of diffusion of the intervention in the same NICU environment, the study was conducted sequentially. In other words, the study included the no lullaby group, Mozart's lullaby group, and mother's lullaby group, in that order, at different periods. Therefore, the infants were assigned to each group in the order of admission.

### Participants

All premature infants were examined before the intervention. The inclusion criteria were as follows: [1] gestational age of 29–35 weeks at birth; [2] after 3 days of postnatal age; [3] infants in an incubator; [4] no feeding intolerance, sepsis, congenital anomalies, or other neonatal complications; [5] no intubation; and [6] consent provided by parents. In this study, the reason for including gestational age of more than 29 weeks was that the auditory structure of the fetus was completed at ~25 weeks and could respond appropriately to auditory stimuli after 28 weeks (38).

This study was undertaken after obtaining approval from the institutional review board (40525-201805-HR-32-04) of K University in Korea. We explained the purpose, methods, and procedure of the study; the confidentiality and anonymity of the data; and the possibility of stopping participation at any time for any reason. Subsequently, written informed consent to participate in the study was obtained from by the parents.

### Intervention Application

The order of activities in this study was as follows: recording of the mother's lullaby, measuring physiological parameters, feeding volume, and body weight before intervention, intervention application, and measuring physiological parameters after intervention. The intervention and all measures were performed at the same time by the same methods every day. In order to record the lullabies sung by the mothers of premature infants, mothers were asked to sing the lullaby while placing a microphone 2–6 inches away from their lips. Each mother was instructed to sing the same song that she often sang to her baby for “Taegyo” during pregnancy. In addition to the lullaby, she was also asked to record activities such as calling her fetus nicknames, and telling fairy tales and stories that were performed as “Taegyo” during pregnancy.

The mother's lullaby was recorded using a recorder, the noise was removed, and the intensity of the sound was adjusted to 45 dB. This criterion was proposed by the American Academy of Pediatrics (AAP) Committee of Environmental Health, and noise levels within the NICU were kept below 45 dB (39). Mozart's lullaby (the minor variation, C major K.265, CJ E&M's lullaby royal Mozart album) was played by adjusting the sound intensity to 45 dB for 15 min. In the control group, the infants were not exposed to any lullaby.

Before providing the interventions, the nurse in charge performed regular nursing activities such as back care, change of position, and diaper changes. All routine nursing care was completed 5 min prior to the intervention, and then the pre-intervention measurest were recorded. To provide a homogeneous environment for the participants, the external environment was controlled by maintaining a constant temperature and humidity inside the NICU and incubator. To minimize light and noise in the NICU, all participants were placed in a nest as a supporting position and the incubator was covered.

During the intervention application in both groups, the mother's lullaby and Mozart's lullaby were played on a speaker in the incubator. No music intervention was performed in the control group. The interventions were applied once a day at 8 pm for 15 min (40) and were conducted for seven consecutive days as described previously (41). This time was selected because there were no examinations or treatments due at that time, making it possible to control the external noise level. The sound intensity was adjusted to 45 dB, and the intensity was checked with a decibel meter. Outcome variables were measured daily while applying the intervention. Physiological parameters were measured twice daily before and after the intervention, and the feeding volume and body weight were measured once before the intervention.

### Blinding

This study was a partially double-blind clinical trial. Although the researcher intervened directly in this study and had information about both groups, the premature infants were unaware of being involved in either groups. During measurement, the infant's physiological parameters were automatically monitored by an electrocardiogram (ECG) before and after the intervention. The researcher obtained data from the monitor before and after 15 min of the intervention once daily. The infants' feeding volume and body weight were assessed by the NICU staff members who were unaware of the assignment of the infants into control and intervention groups. The daily feeding volume was calculated by a blinded physician according to the infants' body weight, status, and previous feeding volume. Measurements of the weight of premature infants is part of the daily nursing activities, and the researchers referred to the data stored in the electronic medical records by the evening duty nurses who used an electronic scale in a blinded way.

### Data Collection

#### Demographic Characteristics of Premature Infants

The demographic characteristics of the participants were sex, gestational age (weeks), birth weight, Apgar score at 1 min and 5 min, delivery type, airway intubation experience, corrected gestational age at the start of the study, body weight, daily feeding volume, and lactation method.

#### Physiological Parameters Measurement

Measured physiological parameters were heart rate, systolic and diastolic blood pressure, respiratory rate, and oxygen saturation. The heart and respiratory rates were measured for 1 min using an ECG monitor (SureSings VM8, Philips, Andover, USA) after attaching a pulse oximetry sensor to the participant during the experimental treatment. Blood pressure was measured by applying a cuff for the ankle of a premature infant and connecting it to an ECG monitor. Measurements were taken with the participants in a stable state with no external stimuli and after all the treatments were performed. In mother's lullaby and Mozart's lullaby groups, the measurements were recorded at 8:00 pm. Music was played for 15 min, and the measurements were taken again at 8:15 pm. Measurements in the control group were also recorded at 8 pm and 8:15 pm daily.

#### Feeding Volume and Body Weight

The infants' feeding volume and body weight were measured as indicators related to the growth of premature infants. The feeding volume consumed at 8:00 pm according to the physician's prescription was used as the feeding volume (unit = cc), which was registered in the electronic medical record by a nurse in charge. The feeding volume was calculated based on the total daily intake, divided into 8 doses, and the feeding volume is the same each time. Body weight (g) was measured at 8:00 pm without the infant's diaper on, using a device installed in the incubator (Omnibed, Giraffe, Wauwatosa, USA).

### Sample Size

**Figure 1:**
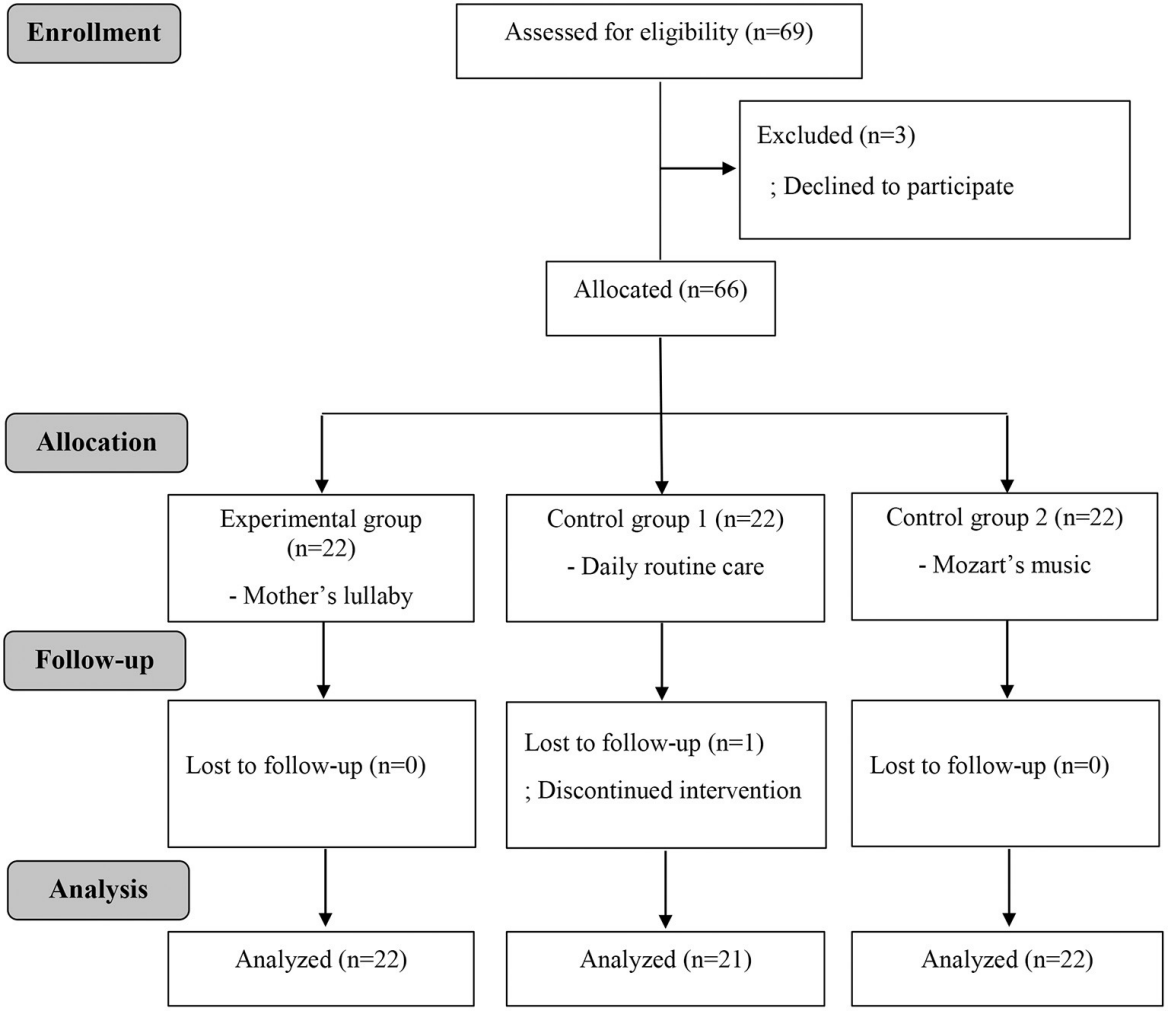
Consort flowchart of the study.

The sample size was calculated using G^*^power version 3.1.9 based on three groups, a significance level of 0.05, a power of 0.80, and an effect size of 0.42 proposed in a meta-analysis related to auditory stimulus (22). According to this analysis, the minimum sample size was 20 participants per group; however, considering the dropout rate, we ultimately recruited 22 participants per group. Initially, the 66 premature infants were divided into no lullaby (control group, 22 infants), Mozart's lullaby (22 infants), and mother's lullaby (22 infants) groups. However, owing to one infant's unstable condition, 65 infants were finally included into this study (Figure 1).

### Statistical Analyses

The collected data were analyzed using IBM SPSS Statistics version 20.0. The general characteristics of the infants were analyzed using means, standard deviations, frequencies, and percentages. The pre-homogeneity test between groups was analyzed using the χ^2^-test, a one-way analysis of variance (ANOVA), and the Kruskal–Wallis test. Systolic blood pressure and respiratory rate were analyzed using the one-way ANOVA (the Scheffe's test was used for the *post-hoc* test). Heart rate, diastolic blood pressure, and oxygen saturation were analyzed using the Kruskal–Wallis test (Mann–Whitney U analysis was used for the post-test). The difference in the values of these physiological parameters between the three groups was analyzed as the average of the difference before and after intervention for 7 days. Changes in feeding volume and body weight were analyzed by repeated-measures ANOVA for 7 consecutive days. A *P* <0.05 was considered statistically significant.

## Results

### Homogeneity Test for Participant's Demographic Characteristics and Dependent Variables

Table 1 shows details of the infants' demographic characteristics and dependent variables. There were no significant differences in sex, gestational period (in weeks), corrected age (in weeks), birth weight (in g), Apgar score (in points), airway intubation experience, and weight. As a result of the homogeneity test for the participant's dependent variable, the heart rate (beats/min) of premature infants in the mother's lullaby, Mozart's lullaby, and no lullaby groups (χ^2^ = 2.70, *P* = 0.075), systolic blood pressure (mmHg) (F = 2.55, *P* = 0.279), diastolic blood pressure (χ^2^ = 1.86, *P* = 0.165), respiratory rate (breaths/min) (F = 0.33, *P* = 0.717), oxygen saturation (%) (χ^2^ = 1.11, *P* = 0.335), feeding volume (cc) (F = 3.15, *P* = 0.050), and the body weight (g) (F = 0.29, *P* = 0.749) were not significantly different.

**Table 1:** Homogeneity test for participant's characteristics between groups (*N* = 65).

**Variables**	**Mother's lullaby group (*n* = 22)**	**Mozart's lullaby group (*n* = 22)**	**No lullaby group (*n*= 21)**	**χ^2^ or F**	***P* value**
	**Mean** **±SD or N (%)**				
Sex					
Female	12 (54.5)	10 (45.5)	10 (47.6)	0.40	0.862
Male	10 (45.5)	12 (54.5)	11 (52.4)		
Gestational age, wk	32.13 ± 2.12	32.56 ± 1.66	32.05 ± 1.86	0.45	0.642
Adjusted for gestational age, wk	33.08 ± 2.17	33.00 ± 1.68	33.71 ± 1.35	1.06	0.354
Birth weight, g	1,618.64 ± 423.62	1,678.18 ± 300.12	1,661.43 ± 462.95	0.13	0.879
Apgar score				0.05	0.949
1 min	6.55 ± 1.60	6.41 ± 1.26	6.43 ± 1.66		
5 min	8.09 ± 1.31	7.91 ± 0.75	7.76 ± 1.34	0.43	0.650
Intubation experience					
Yes	8 (36.4)	6 (27.3)	6 (28.6)	0.50	0.840
No	14 (63.6)	16 (72.7)	15 (71.4)		
Feeding volume, cc per one time	22.91 ± 10.30	20.23 ± 8.93	27.48 ± 9.40	3.15	0.050
Body weight, g	1,675.91 ± 439.25	1,618.64 ± 303.65	1,707.14 ± 405.70	0.29	0.749
Heart rate, beats/min^†^	158.09 ± 17.74	146.41 ± 15.05	151.24 ± 17.42	2.70	0.075
Systolic pressure, mmHg	77.41 ± 5.83	74.18 ± 9.66	74.10 ± 6.97	2.55	0.279
Diastolic pressure, mmHg^†^	45.68 ± 6.79	41.82 ± 7.31	44.05 ± 5.82	1.86	0.165
Respiratory rate, breaths/min	48.82 ± 4.91	47.73 ± 4.87	47.71 ± 5.58	0.33	0.717
O_2_ saturation, %^†^	97.82 ± 1.87	98.55 ± 1.44	98.38 ± 1.75	1.11	

† *Kruskal Wallis test.*

*SD, standard deviation*.

### Changes in Physiologic Parameters, Feeding Volume, and Body Weight

**Figure 2:**
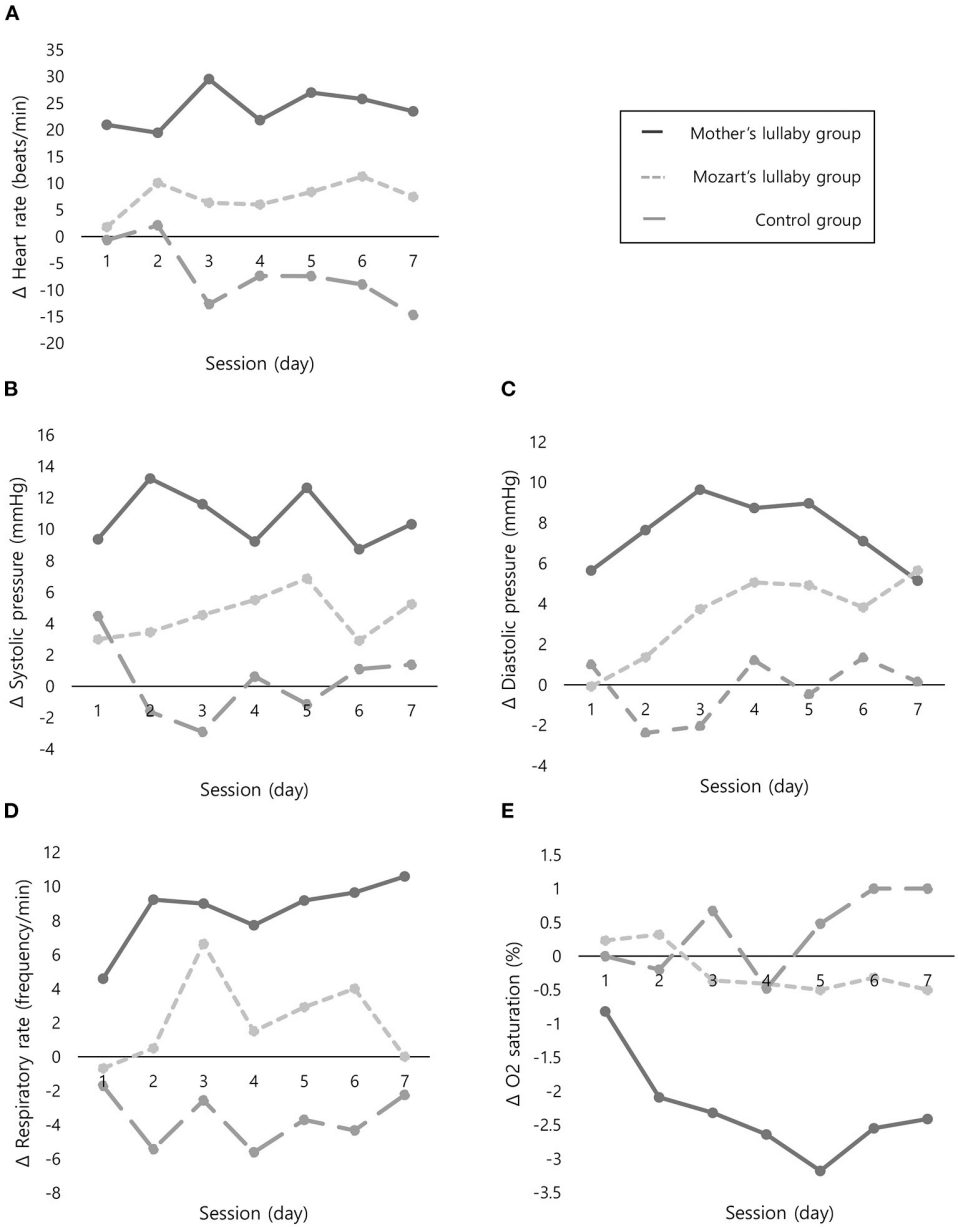
Comparison of changes in physiological parameters before and after intervention between the three groups. **(A)** Mean difference in heart rate. **(B)** Mean difference in systolic pressure. **(C)** Mean difference in diastolic pressure. **(D)** Mean difference in respiratory rate. **(E)** Mean difference in O_2_ saturation. Δ, Mean difference of before and after intervention.

As shown in Table 2, there were significant differences of mean difference of before and after intervention in neonatal heart rate (χ^2^ = 45.03, *P* < 0.001), systolic pressure (F = 43.29, *P* < 0.001), diastolic pressure (χ^2^ = 33.01, *P* < 0.001), respiratory rate (F = 76.06, *P* < 0.001), and O_2_ saturation (χ^2^ = 40.82, *P* < 0.001) between the three groups. As a result of *post-hoc* analysis, the mean difference of the mother's lullaby group and Mozart's lullaby group were both significantly higher than the control group in all physiological parameters (Figure 2). Further, the mother's lullaby group was found to be a significantly higher mean difference than that of the Mozart's lullaby group in heart rate (t = −6.99, *P* <0.001), systolic blood pressure (t = 6.06, *P* <0.001), diastolic blood pressure (t = 5.43, *P* <0.001), respiratory rate (t = 5.66, *P* <0.001), and O_2_ saturation (t = 3.83, *P* <0.001) (data not shown).

**Table 2:** Comparison of heart rate, O_2_ saturation, blood pressure, and respiratory rate between groups (*N* = 65).

**Variables**		**Mother's lullaby group^**a**^ (*n* = 22)**	**Mozart lullaby group^**b**^ (*n* = 22)**	**No lullaby group^**c**^ (*n* = 21)**	**χ^2^ or F**	***P* value**
		**Difference (before–after intervention), Mean** **±SD**				
Heart rate, beats/min	1st session (s)	20.95 ± 18.36	1.77 ± 13.10	−0.67 ± 14.63	45.03, a > b > c^†^	<0.001
	2nd s	19.45 ± 14.40	10.05 ± 12.20	2.10 ± 22.45		
	3rd s	29.50 ± 15.65	6.32 ± 14.82	−12.67 ± 24.17		
	4th s	21.81 ± 14.08	6.00 ± 10.16	−7.38 ± 14.84		
	5th s	27.00 ± 13.89	8.36 ± 12.54	−7.39 ± 17.41		
	6th s	25.77 ± 15.91	11.27 ± 14.19	−9.00 ± 17.62		
	7th s	23.45 ± 15.59	7.45 ± 9.58	−14.71 ± 34.19		
	Mean ± SD	23.99 ± 11.39	7.32 ± 6.05	−7.10 ± 12.58		
Systolic pressure, mmHg	1st s	9.36 ± 5.36	3.00 ± 10.43	4.48 ± 13.	43.29, a > b > c^‡^	<0.001
	2nd s	13.23 ± 6.40	3.45 ± 7.45	−1.62 ± 12.11		
	3rd s	11.60 ± 7.13	4.55 ± 8.71	−2.90 ± 8.31		
	4th s	9.23 ± 16.03	5.50 ± 7.52	0.62 ± 7.87		
	5th s	12.64 ± 5.85	6.86 ± 9.09	−1.14 ± 9.63		
	6th s	8.73 ± 6.46	2.91 ± 7.53	1.10 ± 8.44		
	7th s	10.32 ± 8.52	5.23 ± 10.64	1.38 ± 8.70		
	Mean ± SD	10.73 ± 3.87	4.50 ± 3.74	0.27 ± 3.51		
Diastolic pressure, mmHg	1st s	5.64 ± 6.63	−0.09 ± 9.37	1.00 ± 10.65	33.01, a > b > c^†^	<0.001
	2nd s	7.64 ± 6.90	1.36 ± 6.77	−2.38 ± 9.60		
	3rd s	9.64 ± 5.16	3.73 ± 9.49	−2.05 ± 8.92		
	4th s	8.73 ± 7.28	5.05 ± 8.73	1.20 ± 8.82		
	5th s	8.96 ± 6.70	4.91 ± 9.72	−0.48 ± 8.34		
	6th s	7.09 ± 6.36	3.82 ± 7.27	1.33 ± 7.83		
	7th s	5.14 ± 4.46	5.64 ± 9.51	0.14 ± 8.30		
	Mean ± SD	7.55 ± 3.64	3.49 ± 3.40	−0.18 ± 2.91		
Respiratory rate, breaths/min	1st s	4.59 ± 6.08	−0.68 ± 8.43	−1.71 ± 8.98	76.06, a > b > c^‡^	<0.001
	2nd s	9.23 ± 5.69	0.50 ± 10.94	−5.43 ± 7.95		
	3rd s	9.00 ± 5.35	6.64 ± 12.95	−2.57 ± 7.31		
	4th s	7.73 ± 5.07	1.50 ± 5.33	−5.62 ± 6.68		
	5th s	9.18 ± 5.37	2.91 ± 5.04	−3.71 ±7.40		
	6th s	9.64 ±6.64	4.00 ± 9.98	−4.33 ± 5.51		
	7th s	10.59 ± 7.19	3.50 ± 5.24	−2.24 ± 5.58		
	Mean ± SD	8.56 ± 2.97	2.62 ± 3.93	−3.66 ± 2.69		
O_2_ saturation, %	1st s	−0.82 ± 1.10	0.23 ± 1.66	0.00 ±1.05	40.82, a > b > c^†^	<0.001
	2nd s	−2.09 ± 1.85	0.32 ± 2.08	−0.20 ±2.68		
	3rd s	−2.32 ± 2.30	−0.36 ± 2.04	0.67 ±3.12		
	4th s	−2.64 ± 2.24	−0.41 ± 1.37	−0.48 ±2.06		
	5th s	−3.18 ± 1.79	−0.50 ± 2.27	0.48 ± 2.29		
	6th s	−2.55 ± 1.82	−0.32 ± 2.21	1.00 ± 1.58		
	7th s	−2.41 ± 2.20	−0.50 ± 1.74	1.00 ± 3.00		
	Mean ± SD	−2.29 ± 1.06	−0.22 ± 0.89	0.35 ±0.84		

† *Mann-Whitney U.*

‡ *Scheffe.*

*SD, standard deviation*.

**Figure 3:**
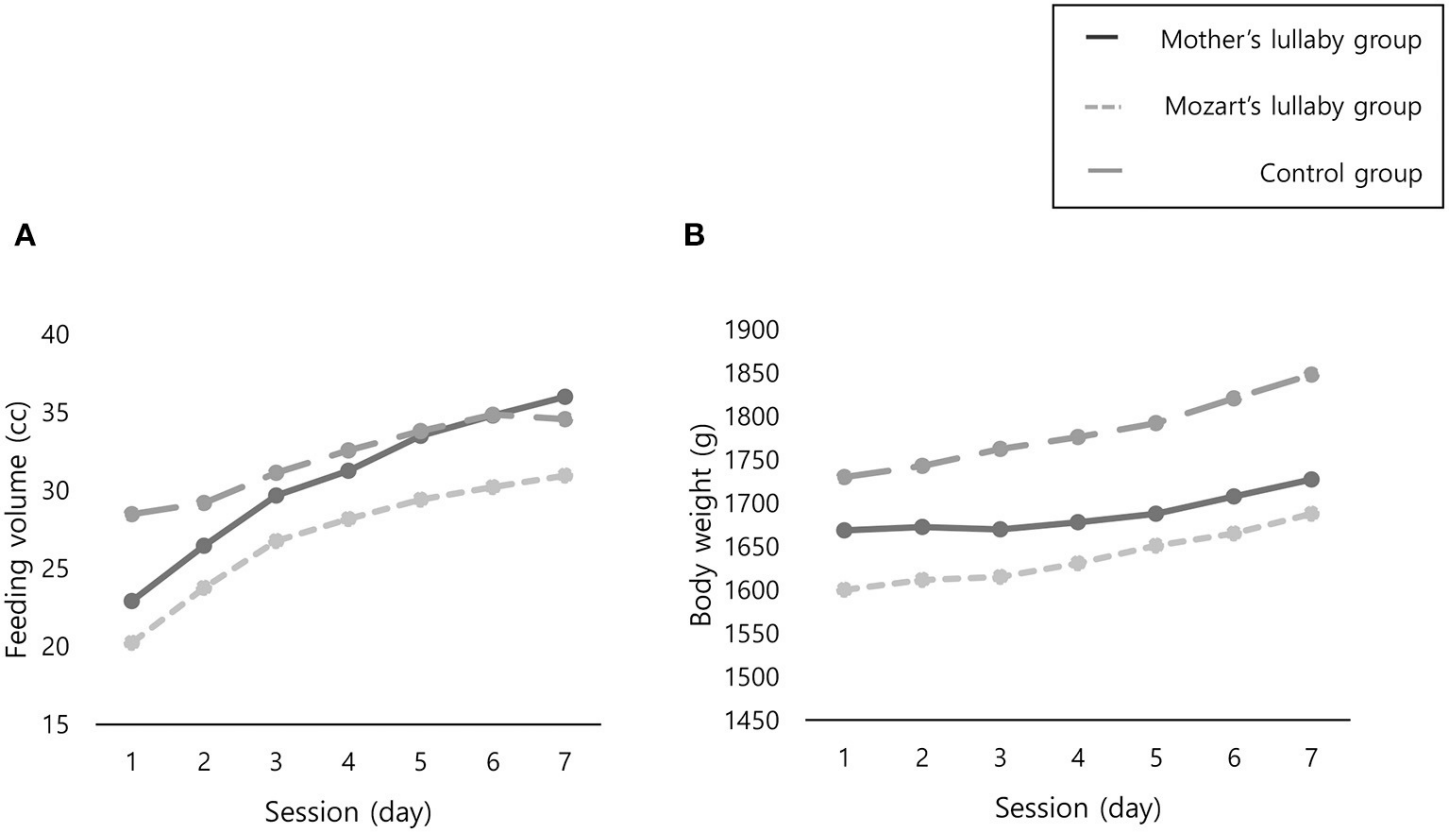
Comparison of changes in feeding volume and body weight between three groups. **(A)** Feeding volume. **(B)** Body weight.

A repeated-measures ANOVA between groups and time as the main factors was used to analyze the feeding volume and body weight (Table 3). There was a significant effect of time (F = 73.22, *P* <0.001) and a significant interaction between time and group (F = 2.46, *P* = 0.041) in feeding volume. However, although body weight had a significant effect over time (F = 43.54, *P* <0.001), there was not a significant interaction between time and group (F = 1.75, P = 0.151) (Figure 3).

**Table 3:** Comparison of the feeding volume and body weight between groups (*N* = 65).

**Variables**		**Mother's lullaby** **group^**a**^ (*n* = 22)**	**Mozart lullaby group^**b**^ (*n* = 22)**	**No lullaby group^**c**^ (*n* = 21)**	**Source**	**F**	***P* value**
		**Mean** **±SD**					
Feeding volume at 8 pm, cc	1st day, d	22.91 ± 10.30	20.23 ± 8.93	28.48 ± 9.2	Group b, c < a Time G * T	1.47 73.22 2.46	0.238 <0.001 0.041
	2nd d	26.45 ± 10.27	23.77 ± 9.65	29.19 ± 9.20			
	3rd d	29.68 ± 11.23	26.77 ± 9.93	31.14 ± 8.71			
	4th d	31.27 ± 11.52	28.18 ± 10.82	32.57 ± 8.36			
	5th d	33.50 ± 11.01	29.41 ± 12.19	33.81 ± 8.33			
	6th d	34.82 ± 11.04	30.23 ± 12.42	34.86 ± 8.82			
	7th d	36.00 ± 10.55	30.95 ± 13.02	34.57 ± 11.11			
Body weight at 8 pm, g	1st day, d	1,668.64 ± 431.91	1,600.00 ± 297.63	1,730.00 ± 400.70	Group Time G * T	0.86 43.54 1.75	0.427 <0.001 0.151
	2nd d	1,672.27 ± 435.26	1,611.36 ± 298.65	1,742.86 ± 370.73			
	3rd d	1,669.55 ± 436.56	1,615.00 ± 300.01	1,762.38 ± 360.80			
	4th d	1,677.73 ± 424.14	1,630.45 ± 298.99	1,776.19 ± 349.22			
	5th d	1,687.73 ± 426.76	1,650.91 ± 296.34	1,791.90 ± 346.64			
	6th d	1,707.73 ± 426.96	1,665.00 ± 296.77	1,820.48 ± 350.62			
	7th d	1,727.27 ± 431.82	1,687.73 ± 292.67	1,848.10 ± 348.66			

G ^*^ T = Interactions between group and time.

## Discussion

We aimed to compare effects of a mother's lullaby and Mozart's lullaby on the physiological parameters, feeding volume, and body weight of premature infants between 29 and 35 weeks of gestation in the NICU. This is because the mother's lullaby is the most common method of prenatal education (Taegyo) during pregnancy in Korea (16, 18), and Mozart's lullaby is one of the most popular classical music pieces used for MT (37). The results of this study have shown that both mother's and Mozart lullabies are effective interventions for physiological homeostasis, but the mother's lullaby was found more significantly effective in physiological stability than Mozart's lullaby for preterm infants. In addition, the mother's lullaby increased the infant's feeding volume than Mozart's lullaby did.

In the literature, physiological parameters such as heart rate, blood pressure, and respiration rate are representative indicators that reflect environmental stress (6, 11, 21–23, 42), and elevated levels of these parameters suggest that they induce energy loss, compromising growth in newborns (9–13). Thus, it was assumed that both types of MTs were effective in relieving environmental stresses in the NICU, resulting in stability of physiological parameters and saving of energy for growth and development. Additionally, physiological stability increases oxygen saturation (43). Conversely, unstable physiological conditions, such as low blood pressure, result in insufficient oxygen levels to carry out normal functions and to promote neurodevelopmental outcomes (44). These results are consistent with the previous studies that used MT (11, 21–23). According to the literature, music can reduce the activity of the sympathetic nervous system and increase activity of the parasympathetic nervous system, thereby stabilizing physiological homeostasis and relaxing the body (43, 45). For these mechanisms, MT has been commonly used to relieve stress (11, 15, 22–24). Regarding to the feeding volume, it correlated with body weight, which is a major parameter of physical growth in infants. In addition, sufficient growth is essential for the development of premature infants. Therefore, the feeding volume ultimately affects the growth and developmental outcomes (34–36). The result of this study that mother's lullaby increase the feeding volume in premature infants is consistent with Blumenfeld and Eisenfeld's finding that mothers' singing improved infant feeding behavior and sucking patterns (46).

Notably, the results of our study showed that the mother's lullaby stabilized the physiological homeostasis and increased the feeding volume of premature infants compared to Mozart's lullaby. These differences can be explained by the mechanism related to mother-child attachmet framework based on “Taegyo”. Overall, MT is known to stabilize physiological parameters by relieving environmental and psychological stress and soothing sympathetic nervous system activity (43, 45). Nevertheless, several previous studies have reported inconsistent results, such as not significantly improving physiological parameters after MT (25, 33), or improving only some of the various physiological measurement variables measured (23, 27, 28). In contrast, this study showed that all four physiological variables–heart rate, blood pressure, respiratory rate, and oxygen saturation– were consistently stabilized after the intervention. In particular, mother's lullaby significantly improved all physiological parameters, showing differentiated results from previous research results. This reals that the lullaby transmitted by the mother's voice is not only familiar to the infants, but also maintains and promotes mother-child attachment even in the NICU (19, 20). According to the existing studies, the mother's voice has been known to strongly affect the formation of the mother-infant attachment, as well as the neurodevelopment of the infant (19, 27). In particular, the maternal lullaby supported maternal and neonatal relationships by promoting emotional closeness and creating early intervention moments (47). This can also be interpreted as the effect of prolonged prenatal care (Taegyo) among Korean mothers.

Until now, there have rarely been reports on the effect of mother's lullaby on the feeding volume of premature infants in NICU, thus, it is difficult to directly compare the results of this study with the ones of previous studies. However, in this study, the possible mechanism of increased feeding volume in the mother's lullaby group might be explained by the physiological stability, which could reduce premature's meaningless activities and energy consumption, and thus could increase feeding volume (48). In addition, the mother's voice acts both as an optimal auditory stimuli, and as an emotional/affective stimulus source for comatose patients in the ICU (49). Emotional and/or familiar auditory stimuli can activate a large network of brain regions, such as the cerebral cortex, thalamus, and limbic system, resulting in improving the physiological function (47, 50, 51). Our possible explanation of the association among the mother's lullaby, physiological parameters, and feeding volume seems related to that the mother's lullaby is proven to have longer effects than classical music (28). For these reasons, the mother's lullaby is likely to have a more positive effect on physiological parameters and feeding volume than Mozart's music.

However, we found that the mother's lullaby did not affect the body weight of premature infants. Previous studies also showed that a mother's voice has no effect on weight gain in preterm infants (36). However, there were conflicting findings regarding significant body weight increase in premature infants after music intervention (52). It should be noted that the weight of premature infants is affected by various factors such as oral intake and fluid therapy, and these exogenous variables cannot be controlled completely (53). Additionally, the participants of our study were premature infants (at least 3 days old). Considering that physiological weight loss may occur up to 7 days depending on the characteristics of the participants, further studies are needed.

To our knowledge, our study is the first to compare the effects of a mother's lullaby and classical lullaby in terms of feeding volume and body weight as well as physiological homeostasis of premature infants in the NICU. Although there were two previous studies comparing mothers' lullaby and classical music were previously reported, they only compared physiological parameters and the outcomes were inconsistent (28, 40). Furthermore, the results of this study have clinical implications. Restricted visiting policies to the NICU had a higher impact on families and were significantly associated with a lack of bonding time and inability to participate in care during the current coronavirus disease pandemic (54). A mothers' lullaby is a suitable auditory stimulation method, and in addition to providing emotional stimulation, it can reduce stress in premature infants and promote attachment to the mother, ultimately promoting development. Moreover, it can be provided without time or place restrictions and is a way to indirectly enable the mother's participation in her child's supportive and developmental care. Therefore, we strongly recommend that mothers' lullaby could be a new alternative intervention to solve the pending issue of neonatal care during the pandemic.

This study had some limitations. First, we did not have complete control over environmental factors, including various external noises in the NICU. Second, this study was a non-randomized clinical trial (RCT) and included premature infants of different gestational ages; therefore, the sample size may not be sufficient to verify the study results. Third, control of confounding variables was not sufficiently performed during the selection of the participants, and small for gestational age, which is related to the weight change of premature infants, is an example of such variables. To validate the findings of this study, RCTs using sufficient participant selection criteria are needed. Fourth, physiological weight loss in premature infants can occur up to 7 days after delivery. Therefore, it is necessary to reconfirm the effect of a mother's lullaby on premature infants after 7 days. Finally, the establishment of an appropriate and safe attachment with the mother is necessary for infants' growth. Therefore, attachment levels should be considered when applying and evaluating the intervention for growth of premature infants.

Despite these limitations, this study was conducted with control of the external environment by applying interventions to the incubator and using consistent values for the parameters. This study can provide substantial data for auditory stimulation methods that could promote physiological homeostasis and growth. Moreover, it is a cost-effective intervention and has several advantages for improving the mothering role and the physiological conditions of premature infants. Therefore, strengthening supportive care, such as playing a mother's lullaby, could be a new alternative to promote the growth of premature infants in the NICU apart from medical approaches.

## Conclusions

The mother's lullaby was found to significantly improve the physiological homeostasis and feeding volumes of premature infants in the NICU. These effects were found to be much more effective than Mozart's lullaby. Moreover, the mother's lullaby can be provided without time or place restrictions and is a way to indirectly enable the mother's participation in her child's care for physiological homeostasis and growth. Therefore, we recommended regular integration of the mother's lullaby into the supportive care of premature infants in the NICU.

## Data Availability Statement

The raw data supporting the conclusion of this article will be made available by the authors on request. Requests to access the datasets should be directed to NK, drkim@kmu.ac.kr.

## Ethics Statement

The studies involving human participants were reviewed and approved by the Ethics Committee of Keimying University. The patients/participants provided their written informed consent to participate in this study.

## Author Contributions

H-JS and NK designed the study. H-JS, H-KO, JP, and NK conducted the research and collected the data. JP and NK coordinated and supervised data collection. H-JS, JP, and H-KO analyzed the data and drafted the initial manuscript. JP, H-KO, and NK reviewed and revised the manuscript. All authors read and approved the final manuscript.

## Funding

This research was supported by the Bisa Research Grant of Keimyung University in 2020. This funder had no role in the study design, data collection, analysis, interpretation, writing the manuscript, or the decision to submit the paper for publication.

## Conflict of Interest

The authors declare that the research was conducted in the absence of any commercial or financial relationships that could be construed as a potential conflict of interest.

## Publisher's Note

All claims expressed in this article are solely those of the authors and do not necessarily represent those of their affiliated organizations, or those of the publisher, the editors and the reviewers. Any product that may be evaluated in this article, or claim that may be made by its manufacturer, is not guaranteed or endorsed by the publisher.
